# Chemically treated groundnut shell fibers for enhanced mechanical and blast performance of concrete

**DOI:** 10.1038/s41598-025-21957-9

**Published:** 2025-10-30

**Authors:** T. R. Praveenkumar, Badrinarayan Rath, Prabhu Paramasivam, Rupesh Gupta, Mitiku Adare Tufa

**Affiliations:** 1 NICMAR Institute of Construction Management and Research, Delhi-NCR, Bahadurgarh, 124507 India; 2 Department of Civil Engineering, Graphic Era Deemed to be University, Dehradun, Uttarakhand 248002 India; 3 Department of Research and Innovation, Saveetha School of Engineering, SIMATS, Chennai, Tamil Nadu 602105 India; 4 Chitkara University Institute of Engineering and Technology, Chitkara University, Punjab, India; 5 Department of Civil Engineering, Salale University, Salale, 245 Fiche Ethiopia

**Keywords:** Concrete, Blast loading, Tensile strength, Finite element analysis, Trinitrotoluene, Engineering, Civil engineering

## Abstract

Groundnut shell fiber, an agricultural byproduct rich in cellulose and lignin, offers potential as a sustainable reinforcement in high-performance concrete. This study investigates the effect of chemically treated groundnut shell fibers on the mechanical and blast performance of fiber-reinforced concrete (FRC). Fibers were subjected to alkali, silane, and acetylation treatments, followed by characterization through tensile testing, FTIR, and XRD analyses to evaluate structural and chemical modifications. The results demonstrated that NaOH treatment enhanced tensile strength and modulus of elasticity due to improved crystallinity and reduced fiber diameter. Concrete specimens incorporating fibers at varying dosages (0.5%, 1.0%, and 1.5% by weight of cement) were prepared and tested for compressive, split tensile, and flexural strengths. Findings revealed that untreated and pretreated fibers generally reduced strength, while post-treated fibers improved performance at optimized levels. The best outcomes were achieved at 0.5% post-treated fiber, showing approximately 10–11% improvement in compressive and tensile strengths compared to the control mix, with flexural strength optimized at 1.0%. Beyond these levels, fiber addition reduced performance due to poor dispersion and higher water absorption. Finite element blast simulations further indicated that treated fiber-reinforced panels exhibited improved stress distribution and reduced localized deformation compared to conventional concrete. Overall, the study highlights that chemically treated groundnut shell fibers can enhance the toughness and energy absorption capacity of concrete when used at optimum proportions. However, the blast resistance results are based on numerical modeling, and further large-scale experimental validation is required before structural application in high-impact environments.

## Introduction

Ecological and the environmental responsibility of the scientific community end in search of green materials to satisfy the demand of high strength materials. The role of the natural fiber is crucial in design of any materials. Green composites possess viable advantages than artificial fibers in terms of weight, cost, handling, properties, and fabrication^1,2^. Thus, in recent days the evolution of the fiber is gaining its paramount growth. Further the natural fibers are renewable and environmentally friendly. At present the application of the fibers are very wide owing to its superior impact resistance, tensile strength, thermal resistance, acoustic properties, and resistance to the fracture^3,4^. Natural fibers are extracted for the fruit, roots, leaf, stem, and other byproducts from the agricultural fields. Since they are obtained naturally, they are harmless, degradable, and available abundant. Fibres are utilized into the concrete in dispersed form because of economic reasons. Steel and polypropylene fibres are most widely used in concrete to arrest the early age cracking. These fibres can act as a secondary reinforcement to enhance the tensile strength of concrete. From the previous studies, major drawback in high performance concrete was found to be early crack formation due to low water cement ratio^5^. Lowering the water content in concrete produces early strength to the concrete specimens. Higher hydration process creates self-desiccation of concrete which ultimately forms cracks in the structure due to volumetric instability^6–8^. The service life of concrete decreases due to early age cracking and solution for the problem is to utilize the internal curing agent in concrete such as wood fibres, natural fibres, water absorbing aggregates, super absorption polymers etc. provides additional required water to concrete during hydration process which prevents concrete from self-desiccation and shrinkage^9^. The mechanical performance of concrete has been studied by using jute and sisal fibres in concrete. The study revealed that jute and sisal fibres can act as an internal curing agent in concrete because of average water absorption capacity. Incorporation of cellulose fibres in concrete enhances the mechanical performance of concrete and withstands the load even after the stress value but the water absorption capacity of cellulose fibres is very low to use as an internal curing agent in concrete^10–12^. Utilizing fibres in concrete enhances the impact resistance, flexural toughness of the concrete specimens. Among the various types of fibres, polymeric fibres has more advantages due to its enhanced durability properties of concrete^13,14^. Fibres were extensively used in pavements and also in other precast elements to arrest shrinkage cracks. It can also as a shear reinforcement in beams^15,16^. Utilization of natural fibres in concrete had great advantage compared to synthetic fibres due to its less environmental impact and economic reasons^17^. Despite advantageous, it should be coated effectively to avoid degradation in alkaline environment^18,19^. Degradation of concrete using natural fibres includes high water absorption, volumetric instability, and alkaline attack. This limitation can be counter attack by modifying the cement matrix and proper coating to the fibres^20^.

The ductility and toughness properties of concrete can be improved by utilizing fibres under variable loading mechanisms. Boulekbache et al. analyzed that orientation of fibres played significant role in improving the flexural strength of concrete. Increase in fiber concrete reduces the workability of concrete, hence the percentage of fibres in concrete should be kept below 3%^8^. Topcu and Canbaz concluded that inclusion of fibres improves the split tensile strength of concrete^21^. Several other researchers reported the enhanced toughness index, tensile strength, and creep behavior of concrete upon inclusion of fibres^22–25^. Addition of steel fibres in recycled aggregates concrete specimens increases the modulus of elasticity of concrete and improves the strength properties of concrete^26–28^. W. Yao et al. investigated that addition of steel fibres beyond 0.5% increases the flexural strength of concrete^29^. The stress pattern observed in the recycled aggregates and fibres incorporated concrete was similar as that of normal aggregates concrete mix^30^. Incorporation of basalt fibres improves the microstructure of concrete in interfacial transition zone and increases the strength properties of recycled aggregate concrete^28,31,32^. Recent studies have shown that incorporating basalt fibers into concrete significantly improves its structural behaviour under adverse environmental conditions, particularly in chloride-rich and marine exposures, where fiber reinforcement enhances durability and resistance to degradation^33^. From the previous studies it is evident that the scope of utilizing natural fibers as a composite material for various applications^34^. Although there was abundant works available on the hybrid concrete however, the addition of the natural fiber such as ground nut shell to the concrete was limited to the author’s knowledge. Besides the chemically treated fiber and the ballistic loading failure are the spot light of the current study. Natural fibers such as coir, sisal, jute, hemp, and banana fibers have been widely investigated as eco-friendly reinforcements in cementitious composites, primarily for enhancing ductility, crack-bridging, and energy absorption. Several studies^35–37^ have reported moderate improvements in tensile and flexural strength, though results are highly dependent on fiber type, treatment method, and dosage. Agricultural residues such as rice husk, sugarcane bagasse, and coconut coir have also been incorporated to improve sustainability while reducing waste disposal problems. However, limited research has focused on groundnut shell fibers, which are abundantly available in many regions but largely discarded as waste. Moreover, prior studies on natural fibers have primarily emphasized static mechanical properties, with very few exploring their role in dynamic or blast resistance. The present study advances this field by not only investigating the effect of chemical treatment on the microstructure and mechanical behavior of groundnut shell fibers, but also by integrating finite element blast simulations to explore their potential in high-performance and impact-resistant concretes. Table 1 shows the comparison of different natural fibers in cementitious composites. For groundnut shell fiber, the concrete evidence base is notably thin compared with other agro-fibers; prior work is largely on fiber treatment and polymer composites, not structural concretes or blast-relevant metrics. The current study directly addresses this gap but should state that further full-scale and real blast testing is needed to establish suitability for high-impact applications.

**Table 1:** Comparison of different natural fibers incorporated in cementitious composites.

Fiber type	Treatment (as tested)	% dosage (basis)	Strength change (28-day unless noted)	References
Coconut (coir)	Often NaOH/“alkali-treated”; some works use latex/silica-fume modified matrices	1–3% (vol./binder)	Compressive: up to + 15.6% at 3%; Split tensile: ~+40% at 2%; gains taper or reverse beyond optimum	^38,39^
Sisal	2.5% NaOH (chemical), 150 °C (thermal), and hybrid (NaOH + thermal)	0.5–2.0% (wt. of cement)	Tensile strength increases for all treatments; Compressive strength increases with thermal/hybrid (prior studies report + 31–45%), but Compressive strength decreases with chemical-only; Flexural strength decreases w.r.t control in that study.	^40^
Jute	Untreated/alkali-treated (varies by paper)	0.5–2.0% (wt. of cement)	Compressive: +15.1% to + 20.3% at 1% (mix-dependent); higher dosages may reduce CS	^41^
Hemp	Untreated short fibers (6–18 mm)	0.125–1.0% (vol., mortar)	Flexural: up to + 10.7% at 0.5%; Compressive: −12.7% to − 28.5% vs. control (workability/voids at higher dosages)	^42^
Kenaf	Chemical treatments incl. NaOH, Na₂CO₃ (varies)	0.25–1.0% (wt./vol., cementitious)	At 1%: Compressive ≈ + 6.5%; Tensile ≈ + 12.7% (composites with kenaf or sisal reported similar optima)	^43^
Sugarcane bagasse fiber (SCBF)	Alkali-treated SCBF	0.5–1.0% (vol./binder)	Compressive: modest increases (≈ + 4% at 1% SCBF, mix with bottom ash); several reports note improvement up to ~ 0.5–1%	^44^

In this study naturally available groundnut shell fiber were collected and subjected to chemical treatment in order increase the tensile properties of fibers. Three different proportions of concrete were casted to analyze the mechanical properties and compared with conventional concrete mix. Pretreated and post treated fiber volume of 0.5%, 1% and 1.5% were incorporated in concrete and tested for strength properties and its resistance to blast loading.

## Materials and methods

### Groundnut shell fibers

Groundnut shell fiber, derived from the outer shells of groundnuts, is an abundant, low-cost, and biodegradable byproduct gaining attention as a sustainable and eco-friendly material. It is primarily composed of cellulose, which provides strength and rigidity; hemicellulose, contributing to flexibility; lignin, adding structural integrity; and minor components such as ash and extractives, which influence thermal properties. With its sustainable properties and wide availability, especially in regions with significant groundnut cultivation, groundnut shell fiber holds great potential for advancing circular and sustainable economies.

### Chemical treatment of ground nut shell fiber

After the collection of ground nut shell, they were cleaned with purified water and allowed to dry in the sun. After that, they were ground and sieved to produce particles with a size of 2 mm. The ground nut shells were soaked in at different concentration of NaOH solution i.e. 4%, 6%, 8% and 10% at the normal room temperature for three hours^45^. After that the groundnut shells were rinsed with distilled water to get rid of any remaining alkali. The ground nut fibers were dried for eight hours at room temperature following again kept in oven for six hours at 100 °C. To create a homogenous solution, 3% oligomeric siloxane by weight was dissolved in a methyl alcohol solution (water: alcohol ratio of 40: 60) and swirled for ten minutes. After being submerged in the solution for three hours at normal temperature, the alkali-pre-treated fibers were cleaned and oven-dried for four hours at 90 °C. The groundnut shell fibers that had been alkali-pretreated were steeped in acetic acid for the acetylation treatment, and then they were treated with acetic anhydride for three hours at room temperature.

### Determination of tensile strength of groundnut shell fiber

To study the properties of groundnut fibers, single tensile fiber tests were conducted on the extracted fibers. The fiber diameters were initially measured using an optical microscope of Carl Zeiss. In this study, 25 samples of both treated and untreated fibers were analyzed. Measurements for each fiber were taken at four different points to ensure accuracy. The mean diameter of the fibers was calculated using statistical tools to minimize measurement uncertainty^46,47^. Tensile testing was performed using a Zwick/Roell universal testing machine equipped with a 50 N load cell. Specially designed rubber jaws provided excellent gripping during the tests. Before testing, any crimp in the fibers was manually removed, and the fibers were mounted on paper frames. Tensile tests were performed at a crosshead displacement rate of 1 mm/min till failure. Over fifty numbers of single fibers of each type were examined in order to produce accurate results. The peak points in the observed stress-strain curves were used to calculate the failure strain and tensile strength. Using Origin software, the modulus of elasticity was determined from the first region of elastic (1–2% strain) of the stress-strain diagram.

### Determination of the density of groundnut shell fiber

Fiber density can be determined using a density gradient tube prepared in a 100 ml graduated cylinder. Mixtures of carbon tetrachloride and xylene were prepared in the following volume ratios (ml/ml): 90/10, 80/20, 70/30, 60/40, 50/50, 40/60, 30/70, 20/80, and 10/90. To create the gradient, 10 ml of carbon tetrachloride was carefully added to 100 ml of xylene, followed by the gradual addition of the nine prepared mixtures. Finally, 10 ml of pure xylene was carefully poured on top. Care was taken to ensure that each mixture did not penetrate more than 12 ml into the previous layer, resulting in a column of liquid with a density gradient ranging from 1.6 g/cm³ at the bottom to 0.87 g/cm³ at the top. The fibers were first wetted in warm xylene and dried. They were then placed on a sheet of paper and teased apart to prevent adherence between fibers. The individual fibers were laid on the liquid’s surface in the cylinder and gently pushed below the surface using a stirring rod. The fibers were allowed to float freely and settle at their respective density levels. After 10–15 min, the position where the fibers floated was recorded, indicating the fiber density.

### Determination of lignin and water content in groundnut shell fiber

For the chemical analysis, parameters such as cellulose, hemicellulose, lignin, wax, ash, and moisture content were measured for both treated and untreated fibers using optimized techniques. The cellulose content was determined following the method proposed in the notable work^48^. The APPITA P11s-7 method was employed to measure the lignin concentration in the fibers. Wax content was analyzed using the Soxhlet extraction process. During the extraction, a mixture of chloroform and ethanol was used as the solvent. The fibers were treated with this mixture for approximately 6 h to extract the wax. After the chloroform dissipated, the wax deposition was collected and its weight measured to determine the wax content. Ash content was evaluated following the specifications outlined in ASTM E1755-61. The moisture content of the fibers was measured using an electronic moisture analyzer. Both treated and untreated fibers were oven-dried for 6 h to remove any residual moisture^49,50^.

1 $$\:\text{T}\text{h}\text{e}\:\text{p}\text{e}\text{r}\text{c}\text{e}\text{n}\text{t}\text{a}\text{g}\text{e}\:\text{o}\text{f}\:\text{t}\text{h}\text{e}\:\text{m}\text{o}\text{i}\text{s}\text{t}\text{u}\text{r}\text{e}=\:\frac{\text{s}\text{a}\text{m}\text{p}\text{l}\text{e}\:\text{w}\text{e}\text{g}\text{h}\text{t}\:\text{b}\text{e}\text{f}\text{o}\text{r}\text{e}\:\text{d}\text{r}\text{y}\text{i}\text{n}\text{g}-\text{s}\text{a}\text{m}\text{p}\text{l}\text{e}\:\text{w}\text{e}\text{i}\text{g}\text{h}\text{t}\:\text{s}\text{f}\text{t}\text{e}\text{r}\:\text{d}\text{r}\text{y}\text{i}\text{n}\text{g}}{\text{S}\text{a}\text{m}\text{p}\text{l}\text{e}\:\text{w}\text{e}\text{i}\text{g}\text{h}\text{t}\:\text{b}\text{e}\text{f}\text{o}\text{r}\text{e}\:\text{d}\text{r}\text{y}\text{i}\text{n}\text{g}}\text{*}100$$

Table 2 represents the detailed mechanical property of the natural fiber.

**Table 2:** Properties of the natural fiber.

Fiber type	Density (g/cm^3^)	Tensile strength (MPa)	Tensile modulus (GPa)
Banana	1.3	500	12
Coir	1.35	101–230	2.5–6.5
Cotton	1.55	300–800	6–12.5.5
Curaua	1.4	90–1010	12.2–97
Flax	1.45	400 − 200	26.5–102
Hemp	1.45	250–950	24–92
Henequen	1.25	430–600	Sep-15
Jute	1.39	320–820	7.1–98
Kenaf	1.41	220–920	14.5–53
Sisal	1.4	360–700	9.0–38

### Fourier transform infrared (FTIR)

The FTIR analysis was performed using a Perkin Elmer Spectrum RXI FTIR Spectrometer, which is capable of capturing 32 scans per minute at a resolution of 2 cm⁻¹. FTIR is a cost-effective, reliable, and straightforward tool for identifying the chemical functional groups and bonds in fibers. The analysis was conducted on both treated and untreated fibers to study their behavior when exposed to NaOH. The chemical functional groups were analyzed over a wavelength range of 400–4000 cm⁻¹. For the measurement process, the fibers were ground into a powder form. The resulting powder was mixed with potassium bromide and pressed under high pressure to form sample pellets. These pellets were tested, and the results were plotted as a graph of infrared light versus wavenumber.

### X-ray diffraction (XRD) analysis

A Seifert XRD-7 diffractometer with an FPM attachment was used to perform X-ray diffraction (XRD) measurements using filtered Cu Kα radiation. Using a thin-film diffraction geometry, a fixed primary beam incidence angle of γ = 5° and a parallel beam setup were used. Crystallographic planes (hkl) formed an angle θhkl − γ with the sample surface; reflections from these planes were recorded in this asymmetric diffraction geometry, where θhkl is the Bragg angle for the (hkl) planes. In the angular range of 10° to 90° in 2θ, pure graphite exhibited a consistent penetration depth of about 75 μm. The use of non-uniform geometry enables the detection of additional diffraction peaks, which is particularly advantageous for materials with weak crystallinity. In contrast, the traditional Bragg–Brentano geometry primarily records 00 L reflections, as it is strongly influenced by the preferred orientation of planes aligned parallel to the surface of the sample. The advantage of the unequal geometry is that it allows for the capture of reflections from crystallographic planes that are oriented at different angles to the graphitic layers.

### Fabrication of composite

Concrete specimens of M40 grade were prepared using locally available materials includes river sand, coarse aggregates passing through 12.5 mm sieve with average bulk density of 1520 kg/m^3^ and ordinary Portland cement of 43 grade were used in this study. The mix proportions of concrete specimens with natural fibers included are shown in Table 3. The control concrete mix specimens is represented by CC, 0.5% added pretreated fibres in concrete specimens by weight of cement is represented by PEF-0.5%, 1.0% added pretreated fibres in concrete specimens by weight of cement is represented by PEF- 1.0%, 1.5% added pretreated fibres in concrete specimens by weight of cement is represented by PEF-1.5%. PSF-0.5% represents 0.5% added post treated fibres in concrete specimens, PSF − 1.0% represents 1.0% added post treated fibres in concrete specimens, PSF − 1.5% represents 1.5% added post treated specimens in concrete specimens. After mixing the pretreated or post treated fibers in concrete the slump cone test was carried and noted in Table 3. Every concrete specimen was cast at room temperature with a consistent water-to-cement ratio of 0.4. Before the casting of concrete cube specimen, the slump value of fresh concrete was conducted and found as 100 mm.

**Table 3:** Mix proportions of natural fibres incorporated concrete specimens.

Sample designation	Cement (kg/m^3^)	Groundnut shell fibre (kg/m^3^)	Water (kg/m^3^)	Coarse aggregate (kg/m^3^)	Fine aggregate (kg/m^3^)	Superplasticizer (kg/m^3^)	Slump height (mm)
CC	445	–	180	1190	698	4.45	75
PEF-0.5%	445	2.225	180	1190	698	4.45	58
PEF − 1.0%	445	4.45	180	1190	698	4.45	45
PEF − 1.5%	445	6.675	180	1190	698	4.45	32
PSF − 0.5%	445	2.225	180	1190	698	4.45	67
PSF − 1.0%	445	4.45	180	1190	698	4.45	56
PSF − 1.5%	445	6.675	180	1190	698	4.45	45

### FEA blast simulation modeling

In this study the blast analysis was performed using the FEA tool. The modeling was done for Concrete-fiber reinforced, atmospheric conditions (air), and TNT. In this study the multifield reference frame (MRF) was employed for better reliability. The total Eulerian meshing nodes were 315, 5200 with ALE algorithm. The concrete placed at the constant standoff distance by varying the TNT weight.

The finite element blast simulation was carried out in ABAQUS/Explicit using a coupled Eulerian–Lagrangian (CEL) framework, which is well established for blast-structure interaction problems. In our model, the Eulerian domain represented the air and TNT, while the Lagrangian domain represented the reinforced concrete panel. This approach allows accurate simulation of shock wave propagation and its interaction with the structure while avoiding mesh distortion issues. Meshing was carried out individually for each part before assembling them in the interaction module for subsequent analysis. The mesh consisted of eight-node linear brick elements (C3D8R) with reduced integration and hourglass control, which are suitable for simulating concrete structures under complex loading. A uniform mesh size of 50 mm was assigned to discretize the concrete, slab, and steel components to ensure computational efficiency and accuracy. In this study, the loads were applied systematically in accordance with IS 875 (Part 1 and Part 2) using ABAQUS/CAE. The dead load (DL) was simulated by defining the material density for concrete (2400 kg/m³), and applying gravity loading in the negative Y-direction using the Gravity Load module in the step definition. This automatically accounted for the self-weight of the slab.

To model the constitutive response of the concrete, plasticity and damage models were employed, which are widely used in high-strain-rate and blast scenarios because they account for strain hardening, strain-rate sensitivity, and thermal softening. The stress–strain relationship (Eq. 2) describes material flow under dynamic loading, while the damage evolution law (Eqs. 3–4) defines failure as a function of accumulated plastic strain, strain rate, stress triaxiality, and temperature. In this study, parameters D₄ and D₅ were neglected, consistent with prior works^43,44^, because temperature dependence was considered minimal under the short-duration blast loading investigated. This assumption has been validated in several published blast response studies, and adopting it ensures computational efficiency without significantly compromising accuracy. The standoff distance was kept constant (300 mm) while varying TNT charge weights (2, 3, and 5 kg) to systematically investigate the effect of explosive magnitude. This design isolates the role of fiber reinforcement under increasing blast intensity while holding geometric boundary conditions constant. The outputs of interest included peak von Mises stress, mid-span deflection, and damage contour evolution, which are standard metrics for blast resistance evaluation.

The Abaqus explicit model was used for the blast of concrete using the TNT. To predict the damage the Johnson-Cook Plasticity model were expressed^50^.

2 $$\upsigma = \left[ {{\text{A}} + {\text{B}}\upvarepsilon _{{\text{p}}}^{{\text{n}}} } \right] + \left[ {1 + {\text{C}}\:{\text{ln}}\left( {\frac{{\upxi _{{\text{p}}} }}{{\upxi _{0} }}} \right)} \right](1 - \uptheta ^{{\text{m}}} )$$

Where, A- Yield stress (For reference temperature and strain); B and C- Coefficient of strain hardening and strain sensitivity rate; n- exponent of strain hardening; m- Thermal softening; $\:{{\upepsilon\:}}_{\text{p}}$ and $\:{{\upxi\:}}_{\text{p}}\:$equivalent plastic strain and plastic strain rate; $\:{{\upxi\:}}_{0}-$Reference strain rate; $\:{\uptheta\:}$-dimensionless temperature.

#### Jonhson–Cook failure model

The entire facture blast modeling was designed based on the continuum damage mechanism, where the Johnson and cook damage model given by

3 $$\upomega = \int_{0}^{{\upvarepsilon _{{\text{f}}}^{{{\text{pe}}}} }} {\frac{{{\text{d}}_{{\text{f}}}^{{{\text{pe}}}} }}{{\upvarepsilon _{{{\text{fl}}}}^{{\text{p}}} ({\text{T}})}}}$$

At $\upomega = 1$, failure occurs.

$\:{\text{d}}_{{\upepsilon\:}}^{\text{p}\text{e}}$ Increment of accumulated plastic strain; $\:{{\upepsilon\:}}_{\text{f}\text{l}}^{\text{p}\text{e}}\left(\text{T}\right)$ Plastic failure envelope

4 $$\:{{\upepsilon\:}}_{\text{f}\text{l}}^{\text{p}\text{e}}\left(\text{T}\right)=\left[{\text{D}}_{1}+{\text{D}}_{2}\text{exp}\left[{\text{D}}_{3}\text{T}\right]\right]\left[1+{\text{D}}_{4}\text{l}\text{n}\frac{{{\upxi\:}}_{\text{p}}}{{{\upxi\:}}_{0}}\right]\left[1+{\text{D}}_{5}{{\uptheta\:}}^{\text{*}}\right]$$

$\:{\text{D}}_{1}$, $\:{\text{D}}_{2},\:\text{a}\text{n}\text{d}\:{\text{D}}_{3}$ are the materials parameters; $\:{\text{D}}_{5}$ Temperature parameter.

The stress triaxiality depends on the temperature and the strain. However in this study $\:{\text{D}}_{4}={\text{D}}_{5}=0\:$ negligible similar to previous studies^51,52^.

## Results and discussion

### Workability of different mixes of concrete

The slump results of Table 3 indicate that the incorporation of groundnut shell fibers significantly affected the workability of concrete. Control concrete (CC) achieved a slump of 75 mm, representing good baseline workability. When pretreated fibers (PEF) were added, the slump decreased sharply with increasing fiber dosage. At 0.5% PEF the slump reduced by approximately 23%, at 1.0% the reduction reached 40%, and at 1.5% the loss was about 57%. This strong decline is attributed to the highly hydrophilic nature of pretreated fibers, which absorb considerable mixing water and increase internal friction within the fresh concrete, leading to reduced flow. In contrast, post-treated fibers (PSF) exhibited comparatively better workability due to silane and acetylation treatments, which reduce hydroxyl groups and water absorption, improving fiber dispersion in the mix. At 0.5% PSF the slump reduction was only about 11% compared to control concrete, while at 1.0% and 1.5% the reductions were 25% and 40%, respectively. Despite being less severe than PEF mixes, the workability loss with PSF still increases at higher dosages because the fibers physically obstruct paste flow and increase viscosity. Overall, the results confirm that workability decreases with fiber addition, but the adverse effect is more pronounced in pretreated fibers than in post-treated ones. The optimum performance in terms of balancing workability and mechanical benefits is achieved with 0.5% PSF, where slump loss remains modest and the mix remains workable.

In addition to the observed slump variations, differences in fiber dispersion and orientation were also noted between the pretreated (PEF) and post-treated (PSF) mixes. In the PEF series, the hydrophilic and roughened surface of fibers caused pronounced fiber clustering and balling, particularly at higher dosages (≥ 1.0%), which not only reduced workability but also introduced localized weak zones within the fresh mix. These clusters tended to trap air, thereby increasing entrapped air content, which is reflected in the significant slump loss and later strength reductions. In contrast, PSF mixes showed better fiber dispersion and more uniform orientation because chemical post-treatments reduced surface polarity and minimized fiber–fiber hydrogen bonding. As a result, clustering was less severe, especially at the optimum dosage of 0.5%, although some orientation irregularities and entrapment of voids still occurred at 1.0% and 1.5%. The presence of randomly distributed fibers in both PEF and PSF mixes also contributed to higher air content compared to control concrete, since fibers interrupted paste flow and hindered full compaction. Overall, these fresh-state observations suggest that post-treatment not only improves interfacial bonding but also plays a critical role in mitigating fiber clustering and air entrapment, thereby making fiber-reinforced concrete more workable and structurally reliable when used at lower dosages.

### Tensile properties of groundnut shell fiber

**Fig. 1:**
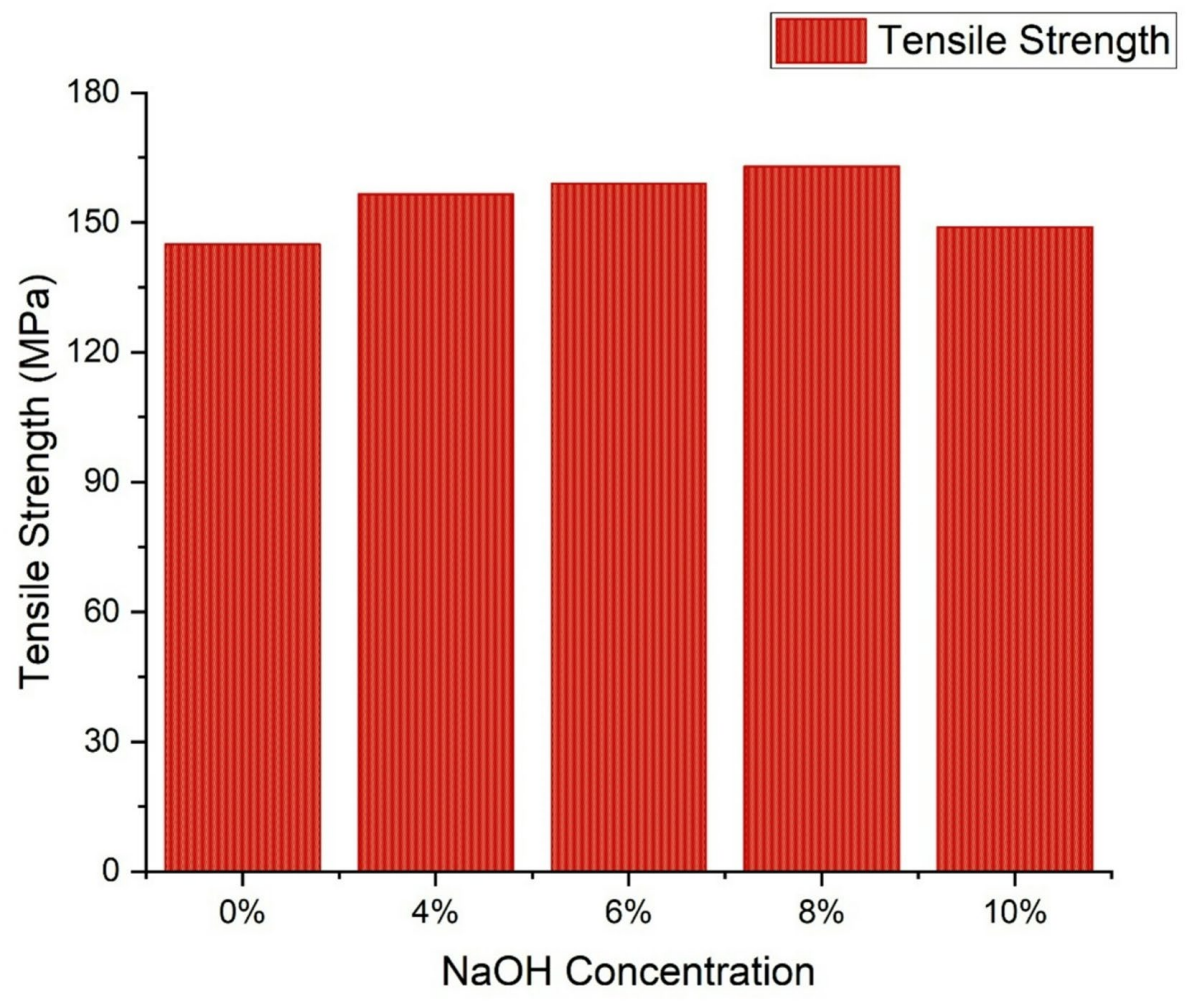
Tensile strength variation of groundnut shell fibers treated with different NaOH concentrations.

The tensile properties of the groundnut shell fiber were evaluated using a single fiber tensile test. The findings unequivocally demonstrate that fibers that have undergone chemical treatment have a markedly increased tensile strength. The samples were tested with NaOH concentrations of 4%, 6%, 8%, and 10%, and the tensile strength of each treated fiber was compared with that of untreated fibers. Determining the optimal NaOH concentration is critical, as the volume of the fiber has a greater influence on the structural strength than does the matrix. To ensure reliable results, 50 single fiber samples, both treated and untreated, were extensively tested. The fiber density decreased from 1.54 g/cm³ to 1.52, 1.48, 1.45, and 1.42 g/cm³ with 4%, 6%, 8%, and 10% NaOH treatments, respectively. Similarly, the fiber diameter significantly reduced with chemical treatment, with the mean diameters of untreated and treated fibers (4%, 6%, 8%, and 10%) measured at 174 μm, 171 μm, 167 μm, and 163 μm, respectively. Figure 1 illustrates the tensile stress–strain behavior of untreated and chemically treated groundnut shell fibers. The results show that untreated fibers possess relatively lower tensile strength and modulus due to the presence of hemicellulose, lignin, and surface waxes, which restrict effective stress transfer. After NaOH treatment, the tensile properties improved significantly, with optimum performance observed at around 6–8% concentration. This enhancement is attributed to the removal of amorphous components such as hemicellulose and lignin, leading to reduced fiber diameter, higher crystallinity, and greater exposure of hydroxyl groups that facilitate better interfacial bonding with the cementitious matrix. Consequently, tensile strength increased to approximately 158 MPa and the modulus to about 18 GPa, indicating stiffer and stronger fibers. Furthermore, the strain at break improved at moderate treatment levels, suggesting better ductility and crack-bridging potential. However, excessive treatment (10% NaOH) caused partial degradation of the fiber surface, resulting in a slight decline in tensile properties. These findings confirm that controlled chemical modification enhances the microstructural integrity and load-bearing capacity of groundnut shell fibers, thereby making them more effective as reinforcement in cement-based composites. The untreated groundnut shell fiber had a tensile strength of 145 MPa and a Young’s modulus of 12 GPa. After NaOH treatment, both properties increased substantially. For example, at 4% NaOH concentration, the tensile strength increased by 8%, and the Young’s modulus improved by 27%. At 6% NaOH concentration, the tensile strength and Young’s modulus reached 158 MPa and 18 GPa, respectively. This significant improvement is attributed to the enhanced crystallinity of the fibers. It is evident that increasing NaOH concentration enhances mechanical properties due to the reduction in fiber diameter, which leads to improved crystalline coating. The removal of hemicelluloses during chemical treatment also contributed to the improved tensile properties. However, at higher NaOH concentrations, such as 10%, a marginal decline in tensile strength and Young’s modulus was observed, likely due to insufficient interfacial bonding between the fiber and the chemical treatment. However, the graphical description indicates that the strain at break improved dramatically with greater concentrations. Based on these results, the ideal concentration of NaOH for chemical treatment to attain good mechanical characteristics is 8%.

### FT-IR and XRD analysis

**Fig. 2:**
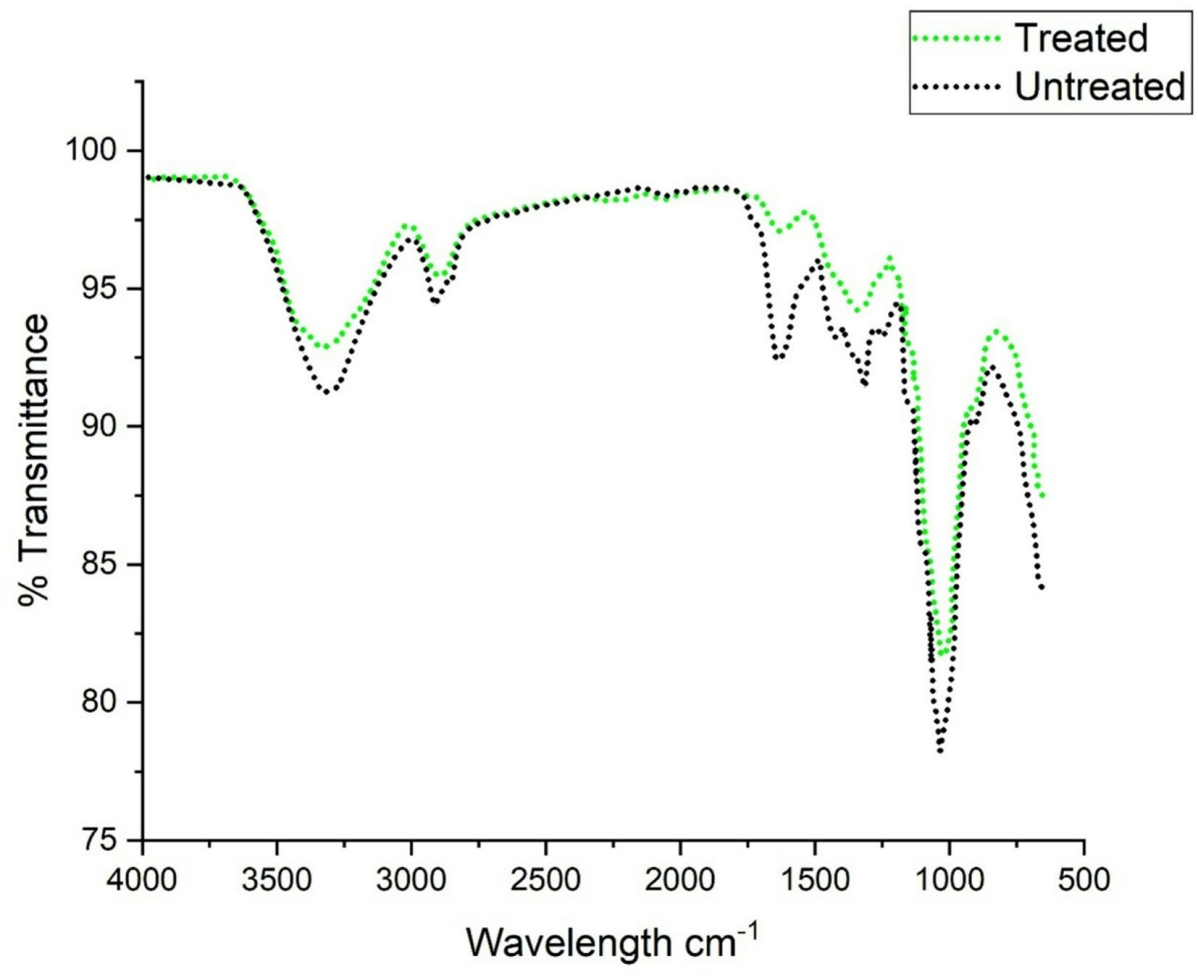
FTIR spectra for both treated and untreated fiber.

Figure 2 presents the FTIR spectra of untreated and chemically treated groundnut shell fibers, revealing the influence of alkali treatment on the functional groups present in the fiber structure. In untreated fibers, characteristic peaks corresponding to hemicellulose, lignin, and waxes are clearly visible, indicating the presence of amorphous and non-cellulosic components that hinder fiber–matrix bonding. After NaOH treatment, notable changes are observed in the spectra: the broad absorption band in the region of 3200–3600 cm⁻¹, associated with O–H stretching vibrations, becomes more intense, signifying increased exposure of hydroxyl groups and improved potential for hydrogen bonding with the cement hydration products. The FTIR spectra for untreated and 4% NaOH-treated groundnut shell fibers, highlighting various transmission bands. After 24 h of NaOH treatment, a significant increase was observed in the peak at 1000 cm⁻¹, corresponding to the -OH group, indicating an enhanced availability of hydroxyl groups for fiber–matrix interface bonding. Additionally, the peak in the range of 3200–3600 cm⁻¹, associated with the interaction of hydroxyl groups and carboxyl groups, also intensified after the 24-hour treatment. The increased intensity of the O–H stretching band (3200–3600 cm⁻¹) reflects greater exposure of hydroxyl groups, which enhances the potential for hydrogen bonding with cement hydration products. Similar increased in intensity were observed for both the 1000 cm⁻¹ and 3200–3600 cm⁻¹ bands in the NaOH-treated groundnut shell fibers. The band around 1000–1050 cm⁻¹, related to C–O stretching of cellulose, also shows higher intensity, suggesting enhanced crystallinity and removal of hemicellulose. The stronger C–O stretching band near 1000–1050 cm⁻¹ confirms improved crystallinity and higher cellulose content, while the reduction of peaks around 1730 cm⁻¹ and 1240–1260 cm⁻¹ demonstrates the partial elimination of hemicellulose and lignin. Furthermore, a reduction in peak intensity near 1730 cm⁻¹, which corresponds to C = O stretching in acetyl and uronic ester groups of hemicellulose, confirms their partial elimination during treatment. Similarly, the diminished absorbance in the 1240–1260 cm⁻¹ range indicates cleavage of lignin-related bonds. These spectral modifications demonstrate that chemical treatment effectively removes amorphous constituents and enriches the cellulose content, thereby increasing the fiber’s compatibility and interfacial bonding capacity with the cement matrix. Such microstructural improvements directly support the enhanced tensile properties and strength performance observed in the treated fiber-reinforced concrete specimens. Also, These changes collectively improve the fiber’s compatibility and interfacial bonding with the cementitious matrix, thereby contributing to enhanced mechanical performance of the composite.

**Fig. 3:**
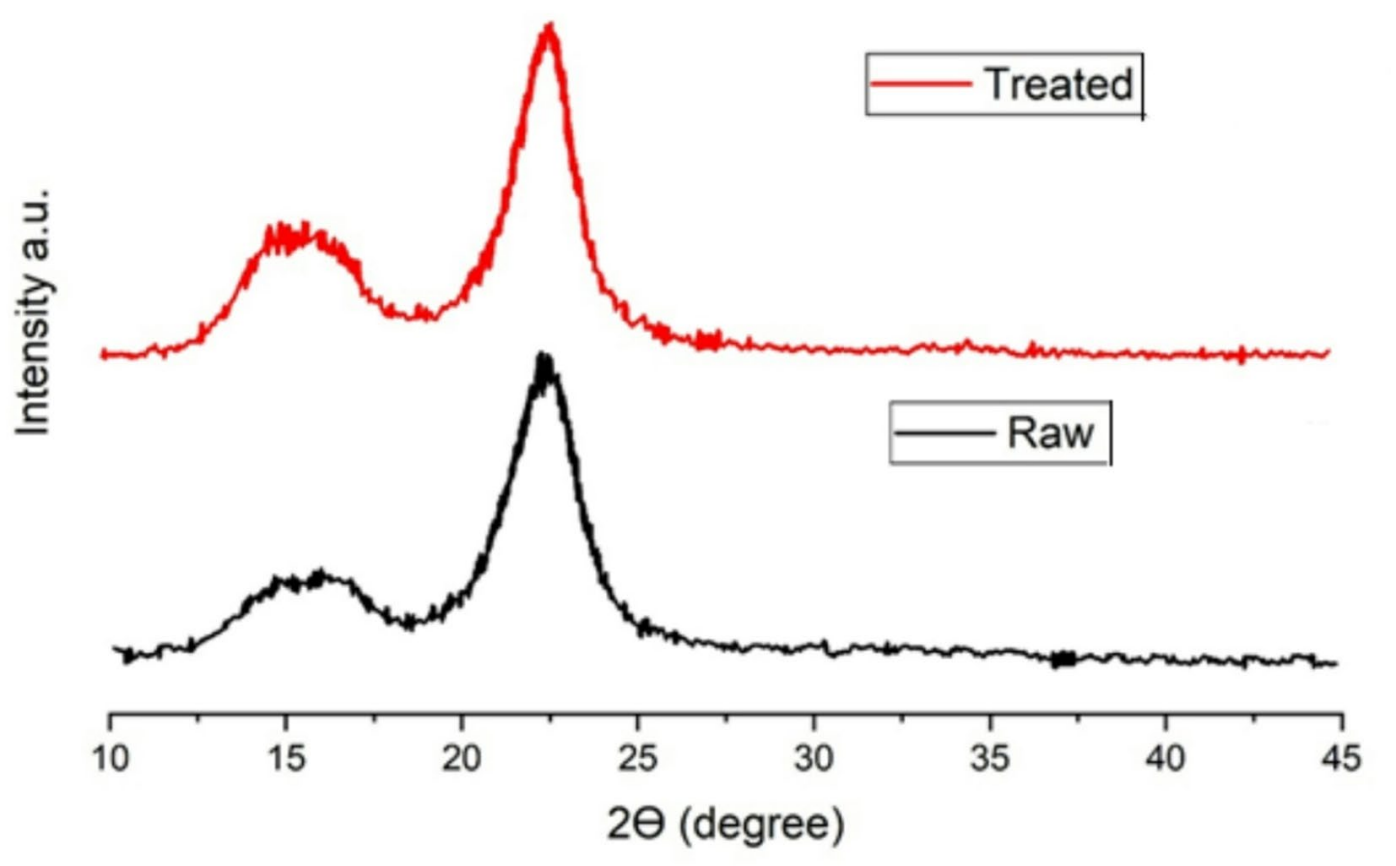
XRD spectra for both treated and non-treated fiber.

Figure 3 illustrates the XRD patterns of untreated and chemically treated groundnut shell fibers, highlighting the structural modifications induced by alkali treatment. In untreated fibers, the diffraction peaks appear broader and less intense, which is characteristic of a higher proportion of amorphous phases such as hemicellulose and lignin. These amorphous constituents contribute to weaker mechanical behavior and reduced dimensional stability. After NaOH treatment, the XRD profile shows sharper and more intense peaks, particularly around 2θ = 22°, which corresponds to the crystalline cellulose (002) plane. This increase in peak intensity and reduction in peak width indicate enhanced crystallinity and improved ordering of cellulose microfibrils. The XRD differences between untreated and treated groundnut-shell fibers arise from alkali-induced microstructural changes in the lignocellulosic matrix. NaOH removes amorphous constituents (hemicellulose, part of lignin and surface extractives), which suppresses the broad amorphous halo and makes cellulose reflections sharper and more intense—i.e., a higher apparent crystallinity and lower peak full-width at half-maximum due to larger/more ordered crystallites (cellulose microfibrils). Mercerization also increases inter-fibrillar spacing and promotes microfibril alignment, improving coherent scattering along cellulose planes (commonly near the cellulose (002)/(200) family), hence the observed peak sharpening/intensity gain after treatment. At overly high NaOH levels, partial peeling and chain scission of cellulose can occur, reducing crystallinity and slightly broadening or weakening peaks again—consistent with a decline after “over-treatment.” The removal of hemicellulose and partial delignification reduces the amorphous background, thereby exposing the crystalline regions more prominently. The improvement in crystallinity enhances the stiffness and tensile strength of the fibers, as cellulose provides the main load-bearing capacity. However, at very high concentrations of NaOH, a slight reduction in crystallinity may occur due to surface damage or partial degradation of cellulose chains. Overall, the XRD results confirm that chemical treatment strengthens the structural integrity of the fibers by enriching crystalline cellulose, which directly supports their improved performance when incorporated into cement-based composites.

The appearance of many peaks in the diffraction patterns generated using asymmetric diffraction geometry during the experiments could be explained by the comparatively high angular width values of the orientation distribution functions in the examined samples. Notably, the annealing temperature affected the peak shape and breadth of these patterns. The degree of crystalline structural ordering and the size of coherent domains were the key factors associated with the broadening of X-ray peaks. Improved atomic ordering, a larger domain, or both could be the outcome of annealing. In particular, it was anticipated that annealing would improve the (00 L) layer structure in the groundnut shell fibers, frequently in conjunction with a decrease in micro strain. The peaks of pure graphite were represented by labeled indices in the diffraction patterns are displayed in Figs. 2 and 3. The Fig. 2 shows that the increase in the –OH peak around 1000 cm⁻¹ after NaOH treatment indicates removal of hemicellulose and exposure of hydroxyl groups, which improves chemical bonding with the cement hydration products. This explains why post-treated fibers showed better strength than untreated ones. From Fig. 3 it can be noticed that the sharper and more intense peaks after treatment confirm increased crystallinity, which enhances fiber stiffness. This correlates with higher tensile strength and modulus values observed in Table 1 and later reflected in concrete mechanical tests. Hence it can be concluded that, the chemical changes identified by FTIR and XRD are directly responsible for the improved bond strength and mechanical performance of concrete containing treated fibers. The 002, 100, and 110 peaks were regularly seen in these patterns, with the 004 and 006 peaks occasionally showing up as lesser ones.

The diffraction profiles of the composite samples annealed at 2800 °C revealed significant structural changes. Computational simulations indicated an increase in the lateral size of the carbon sheets and a notable reduction in lattice spacing fluctuations within the planes, which primarily accounted for the unusual shapes of the 100 + 004 and 110 + 006 peak pairs. Furthermore, these changes were associated with a stronger preferred orientation of the (00 L) planes. In groundnut shell fiber, the (00 L) layers were randomly oriented within coherent regions along the c-axis, as reflected in the asymmetry of the 100 and 110 peaks. However, greater care was required in analyzing the composites, as the resulting diffraction patterns represented a combination of contributions from both the fibers and the matrix, which constituted approximately 20% of the samples.

### Mechanical properties of the fiber reinforced concrete

**Fig. 4:**
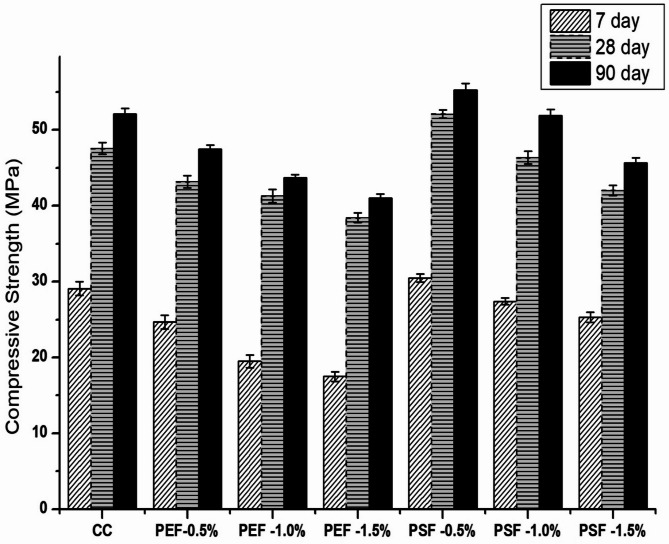
Compressive strength of the fiber reinforced concrete.

Figure 4 shows the compressive strength of groundnut shell fibre incorporated concrete specimens tested at 7, 28 and 90 days. At 7 day compressive strength of 0.5%, 1% and 1.5% pretreated fibres incorporated concrete specimens showed 15.1%, 32.3% and 39.8% lesser compressive strength respectively as compared with control concrete mix (CC), whereas 0.5% post treated concrete specimens showed 5% higher compressive strength as that of CC, 1.0% post treated concrete specimens showed similar strength as that of CC and 1.5% post treated specimens showed 13% reduction in compressive strength as that of CC (Table 2). Chemical treatment of fibers improves the compressive strength of fibres significantly. At higher curing period i.e. 28 and 90 days, strength of 0.5% post treated specimens showed higher compressive strength than other concrete specimens. The increment in strength of 0.5% post treated specimens were found to be 10% and 20% higher than control mix and 0.5% pretreated concrete specimens respectively. The observed improvements at optimum dosage are in line with other NaOH-treated natural fibers such as sisal, coir, and hemp, which also showed increased strength at low contents but reductions at higher dosages^53^. However 1% post treated specimens showed approximately similar strength gain as that of control mix (CC). A simple comparison between 1% pretreated and post treated fibers in concrete depicts that post treated fibers inclusion in concrete showed 7% and 10% increase in strength as that of pretreated specimens at 28 and 90 days respectively. Incorporation of 1.5% natural fibres in concrete had shown significant reduction in strength on both pretreatment and post treatment process at all the curing ages. The maximum replacement of fibers in concrete was found to be 1% by weight of cement, beyond the replacement of fibres reduces the compressive strength of concrete. The maximum strength was achieved in 0.5% post treated fibres incorporated concrete specimens. Incorporation of fibres in concrete acts as a internal curing agent provides additional water during hydration process and enhances the strength of the concrete. A fiber inhibits the self desiccation of concrete owing to low water cement ratio. The reduction in strength of pretreated fibers incorporated concrete was mainly due to high water absorption property of fibers which makes the concrete to slower down the hydration process. Proper chemical treatment of fibers avoids the degradation of fibers due to alkaline attack. Treating the fibres with 4% NaOH enhances the stiffness and toughness index of specimens, thereby enhances the strength properties of concrete specimens. The results obtained from this study had good agreement with the study made by Shehryar Ahmed and Majid Ali^37^.

**Fig. 5:**
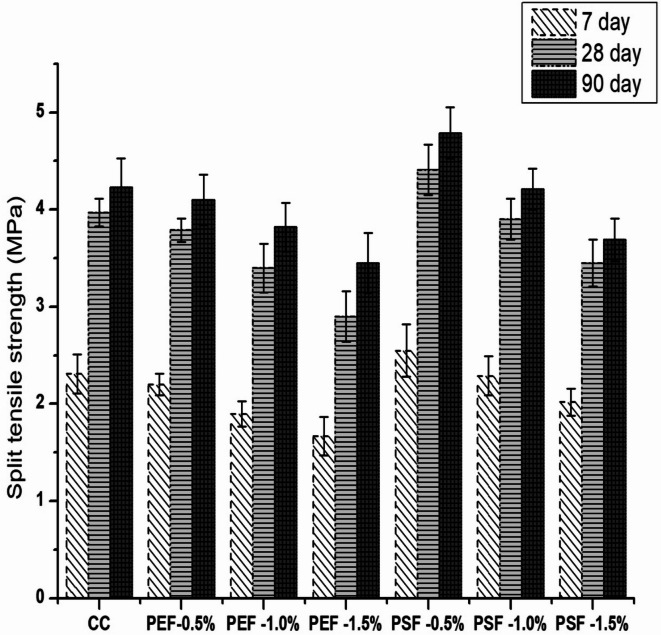
Split tensile strength of the fiber reinforced concrete.

Figure 5 represents the split tensile strength of groundnut shell fibre incorporated concrete specimens tested at 7, 28 and 90 days. The results of split tensile strength achieved quite similar to the results obtained from compressive strength. The replacement of 0.5% fibers in concrete without any chemical treatment reduces the split tensile strength of concrete. At 28 days of curing period, the strength of 0.5%, 1% and 1.5% pretreated fiber inclusion in concrete reduces the splitting tensile strength by 4.5%, 14% and 26.9% respectively as that of control concrete mix. In contrast, the tensile strength of concrete with 0.5% of post-treated fiber incorporation was improved by 10.3%, 11%, and 12.7% at 7, 28, and 90 days, respectively, compared to the control concrete mix. The splitting tensile strength of PEF-0.5%, PEF-1%, PEF 1.5%, PSF 0.5%, PSF-1% and PSF-1.5% at 7 days were 2.2, 1.9,1.67,2.55,2,29 and 2.02 MPa respectively as compared to 2.31 MPa of control concrete mix. As discussed above the presence of fibers in concrete generates additional water in hydration process of high strength concrete and stops the degradation of concrete due to autogenous shrinkage. The optimum content of fibres inclusion in concrete was found to be 0.5% treated chemically and beyond increase in fibers does not gain significant improvement in strength.

**Fig. 6:**
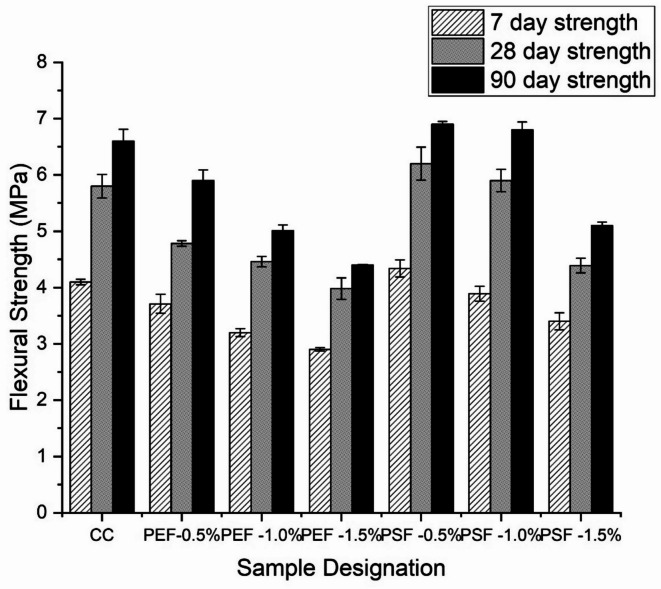
Flexural resistance of the fiber reinforced concrete.

Figure 6 shows the flexural strength of ground nut shell fiber incorporated concrete specimens tested at 7, 28 and 90 days. The flexural strength of post treated fibers had not shown any improvement in strength at early ages. However at later stages, incorporation of fibers after chemical treatment shows promising results as compared to control concrete mix. In comparison to the traditional mix, the flexural strength of 1% post-treated fibers increased by 2% and 3% at 28 and 90 days, respectively. However, adding 1% post-treated fibers produced results in split tensile and compressive strength that were comparable to those of a standard blend. Specimens that were 0.5% post-treated exhibited the maximum flexural strength when compared to untreated specimens. The improvement in strength of 0.5% post treated incorporated specimens were found to be 7%, 29%, 39%, 49%,9% and 41% higher strength as compared to CC, PEF-0.5%, PEF-1%, PEF 1.5%, PSF-1% and PSF-1.5% respectively after 28 days of curing. The optimum percentage of fibers inclusion in concrete for flexural strength of concrete was found to be 1%, beyond its addition leads to degradation of concrete. The increase in strength observed in fiber-reinforced concrete incorporating post-treated groundnut shell fibers can be attributed to the combined effects of improved fiber–matrix interaction, controlled fiber dispersion, and the internal curing action of the fibers. Chemical treatment with NaOH, followed by silane and acetylation, removed surface impurities and reduced hydrophilicity, thereby exposing hydroxyl groups and enhancing crystallinity. This modification not only increased the tensile strength and stiffness of the fibers themselves but also promoted stronger interfacial bonding with the cement hydration products. At the optimum dosage of 0.5%, fibers were well distributed within the matrix, effectively bridging microcracks and restraining their propagation under load. This crack-arresting mechanism improved both tensile and flexural strengths and contributed to higher compressive resistance by delaying crack coalescence. Additionally, the porous structure of the fibers allowed them to act as internal reservoirs of water, releasing moisture gradually and enhancing the hydration of cement in low water–cement ratio mixes. Together, these mechanisms explain why chemically treated groundnut shell fibers significantly improved the mechanical properties of concrete at optimal contents, while untreated or excessive fibers reduced performance due to poor bonding, clustering, and higher water absorption.

Figures 4, 5 and 6 show treated fibers improve mechanical performance, while untreated/over-dosage reduce it, directly supporting the study’s goal of optimizing fiber treatment and dosage. From Fig. 4, it can be observed that at 28 days, 0.5% post-treated fiber (PSF-5%) achieved ~ 10% higher compressive strength compared to control (CC), while higher fiber dosages (1.0% and 1.5%) reduced strength. This shows an optimum dosage exists. Similarly from Fig. 5 it can be noticed that at 28 days, PSF-5% showed ~ 11% higher tensile strength, while PEF mixes (pretreatment only) reduced strength by 5–27%. Also, Fig. 6 itself explains that The maximum flexural strength was achieved with PSF-10%, giving ~ 3% improvement at 90 days compared to CC.

**Fig. 7:**
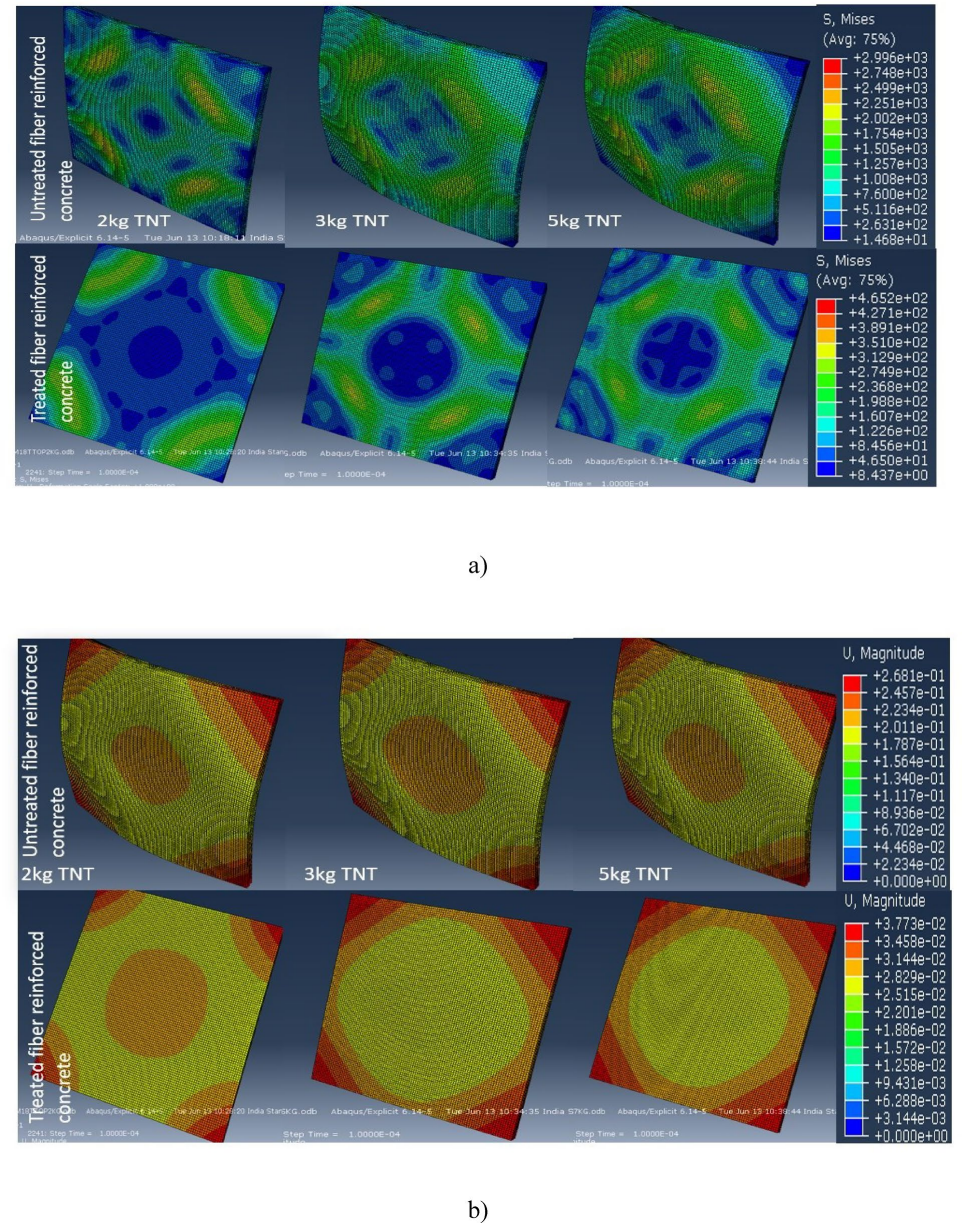
Numerical results of concrete undergone blast loading for treated and untreated fiber reinforced concrete.

**Fig. 8:**
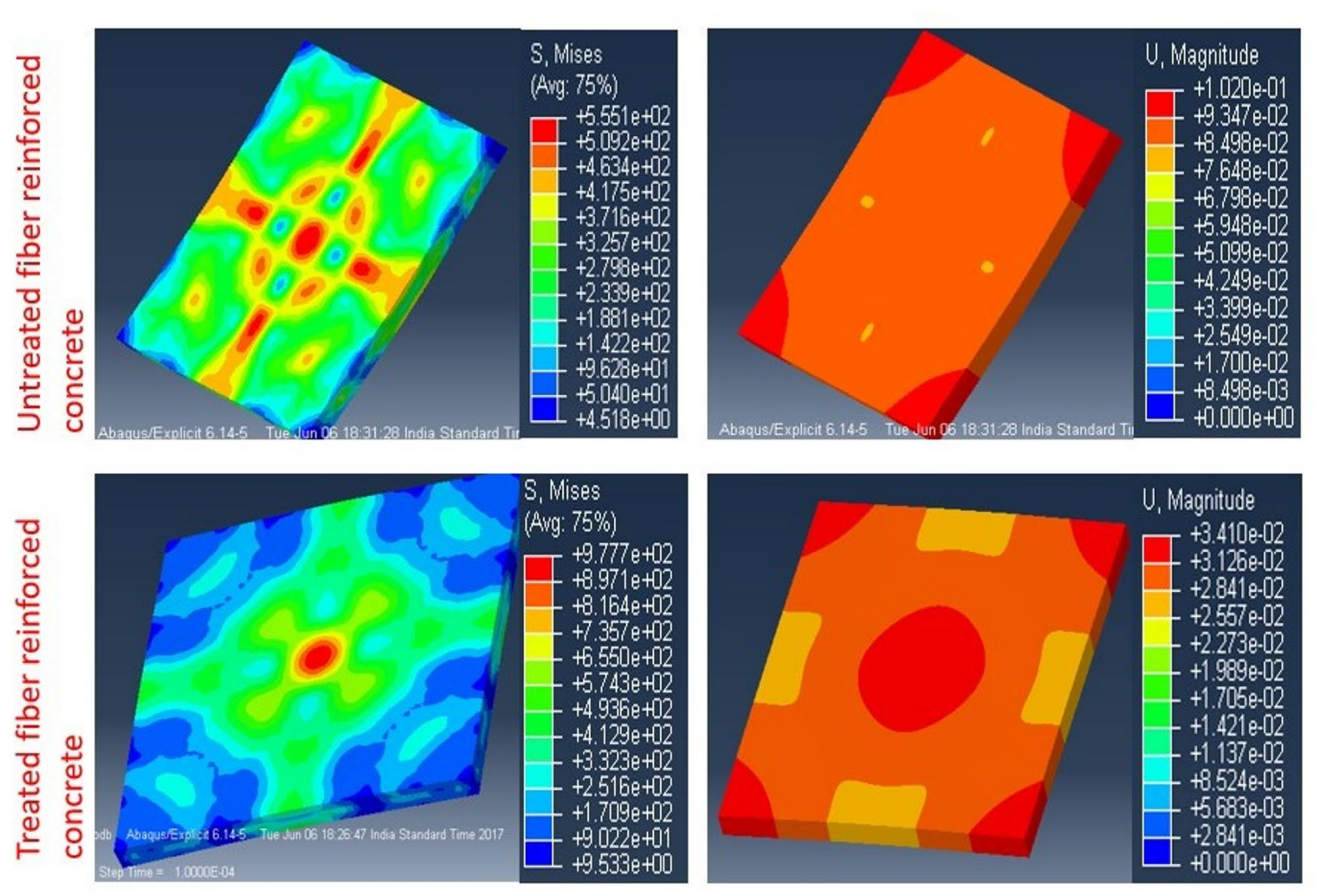
Blast loading using FEA.

The FTIR (Fig. 2) and XRD (Fig. 3) analyses confirm that chemical treatment significantly alters the structure of groundnut shell fibers, enhancing crystallinity and exposing hydroxyl groups that improve fiber–matrix bonding. These structural modifications are directly reflected in the mechanical results. As shown in Fig. 4, 0.5% post-treated fiber (PSF-5%) achieved about 10% higher compressive strength than the control mix (CC), while higher dosages reduced strength due to poor dispersion and excess water absorption. Similarly, Fig. 5 indicates that PSF-5% improved split tensile strength by ~ 11%, whereas untreated and pretreated fibers lowered performance. Flexural strength trends (Fig. 6) further confirm that 1.0% post-treated fiber gave the best improvement at later curing ages. The blast simulation results (Figs. 7 and 8) support these findings: treated fiber-reinforced panels showed lower peak stresses (reduced from ~ 200 MPa in control to ~ 35 MPa) and smaller deflections, demonstrating enhanced energy absorption. These outcomes clearly link the microstructural modifications observed in FTIR/XRD to the improved macroscopic mechanical and blast-resistant behavior of the composites.

### Blast loading analytical results

In this study the blast analysis was performed using the finite element method. The concrete was modeled and the properties were assumed based on the experimental outcomes. The concrete panels were discretized into small numbers of elements using lagrangian mesh. The edges are assumed as the rigid materials to visualize the impact and the friction coefficient was 0.3. To avoid the zero energy models the hourglass control was employed. The similar control was validated from the previous work^54,55^. From the results, the model with the fiber contains higher plastic strain rate which signifies the distribution of the stress throughout the panel compared to conventional concrete. Addition of the fiber to the concrete increases the distribution rate of both stress and strain. In the simulation the TNT placed at constant standoff distance of 300 mm. The stress and the displacement contours are obtained for the concrete model which exposed to 2 kg, 3 kg and 5 kg of TNT^56^. At 2 kg of TNT, the deflection and the stress produced are very low. Figure 7 displays the ABAQUS/Explicit finite-element results for concrete panels subjected to near-field air-blast, comparing untreated/conventional concrete with panels containing post-treated groundnut-shell fibers. The model uses a coupled Eulerian–Lagrangian/ALE setup with a multi-physics reference frame: the panel is meshed in Lagrangian coordinates, the surrounding air/TNT domain in Eulerian coordinates, and hourglass control is applied to avoid spurious zero-energy modes; boundary friction is taken as 0.3. Loads are imposed via TNT charges at a constant 300 mm stand-off with cases analyzed at 2, 3, and 5 kg. The plotted contours (parts a–b) show that, at 2 kg TNT, global deflection and stress are comparatively low, while higher charges increase response severity. Critically, the treated-fiber panel develops broader plastic-strain fields (i.e., stress/strain redistribution) but exhibits lower peak von Mises stress and smaller out-of-plane deflections than the untreated/conventional panel, indicating better energy absorption and mitigation of localized damage. Quantitatively, the simulations report peak stresses of ~ 200 MPa for non-treated/conventional concrete versus ~ 35 MPa for treated-fiber panels under comparable loading, consistent with improved crack-bridging and toughness at the optimized fiber dosage. These results support the mechanistic link between fiber treatment and enhanced impulsive resistance; however, they are model-based, specific to the analyzed charge/stand-off combinations, and should be interpreted as indicative trends pending experimental blast validation. Besides, addition of the fiber increases the load distribution, thus the deformation is negligible compared to conventional concrete. The maximum stress exposed by the both non-treated and treated were 200 MPa and 35 MPa. As a result, adding fibers increases the concrete’s impact strength. Figure 8 synthesizes the finite-element blast analyses to show how the panel response varies with fiber content at a fixed stand-off (≈ 300 mm) and selected TNT masses (2, 3, and 5 kg). It complements the contour views of Fig. 7 by plotting response metrics—principally peak stress/damage indices and maximum out-of-plane deflection—so trends are explicit. The key trend is non-monotonic: panels with an optimized, post-treated fiber dose (~ 0.5%) exhibit lower peak stresses and smaller deflections than control concrete (improved stress redistribution/energy absorption), but increasing the fiber volume beyond this (≥ 1.0–1.5%) reverses the benefit, producing larger deflections and earlier damage under the same impulse. Mechanistically, the model reflects two competing effects captured by the constitutive formulation: (i) treated fibers enhance crack bridging and spread plasticity (beneficial), while (ii) excess fibers degrade matrix continuity/raise void content and stiffness loss, so the impulsive response becomes more compliant and local demands grow (detrimental). The figure also shows the expected load-severity scaling—all response measures increase as TNT mass rises—while preserving the “optimum-dose” ordering across masses. Together, these curves operationalize the paper’s message: post-treatment helps, but only at a limited dosage; more fiber is not always better. Note that these findings are model-based (Abaqus/Explicit with ALE air domain and Johnson–Cook plasticity/failure in the concrete), tied to the chosen parameters and single stand-off; they should be viewed as indicative until validated by physical blast tests or a full sensitivity study. To understand further, the concrete tested for 3 kg and 5 kg of TNT. In both cases, the treated concrete exhibits superior strength compared to the non-treated fibers. Further it represents the distribution of stress and displacement when the fiber volume increases. As the fiber volume increases, the strength of concrete was reduced. The reduced stress and deflection values from simulations (Figs. 7 and 8) indicate that post-treated fibers help dissipate blast energy more effectively, aligning with the research goal of improving impact resistance.

The Fig. 8 depicts the reduction of the concrete strength. From the analysis it is understood that, addition of the fibers to the concrete increases the strength of the mechanical structure. On the contrary, as the volume of the fibers increased further, the strength and the stiffness of the concrete is reduced. Thus it is evident that choosing an optimized fibers volume is crucial. This study is reference of the future work based on hybrid concrete.

While the results demonstrate promising improvements in mechanical performance and simulated blast resistance, several limitations should be considered when interpreting these findings. The blast analysis was restricted to numerical simulations, which, although validated against literature, cannot fully replicate real explosive events. In addition, the mechanical testing was carried out on a relatively small number of laboratory-scale specimens, and no full-size panels or structural members were assessed. This scale effect may influence the practical applicability of the results. The observed improvements with 0.5% post-treated groundnut shell fibers (≈ 10–11% increase in compressive and tensile strength at 28 days) are comparable to or slightly lower than the strength gains reported for coir and sisal fibers at similar dosages (10–15%). However, the reduction in strength at higher fiber contents (≥ 1.0%) parallels findings by Saulo et al. (2018)^57^ for jute and hemp, indicating that overdosing of natural fibers generally compromises matrix integrity due to poor dispersion and water absorption. While most prior studies on NFRC have primarily focused on static mechanical properties^58^, the present work extends this understanding by incorporating blast simulations to evaluate dynamic response. Unlike most prior agricultural fibers, groundnut shell fibers exhibited a marked increase in tensile modulus after alkali treatment (≈ 18 GPa at 6% NaOH), which directly translated into better crack-bridging efficiency. Importantly, while previous studies have largely focused on static loading, the present work extends the scope by incorporating blast simulations. The reduction of peak stress from ~ 200 MPa in control concrete to ~ 35 MPa in treated-fiber panels under 5 kg TNT load suggests that groundnut shell fibers can provide effective energy dissipation under dynamic loading, a contribution not widely reported for other agricultural fibers. This comparison underscores the novelty of using chemically treated groundnut shell fibers not only as a sustainable additive but also as a potential candidate for impact-resistant applications. Furthermore, the long-term durability of chemically treated groundnut shell fibers in concrete was not investigated, which is critical for assessing performance under real service conditions. Finally, the blast simulations were performed with only a few TNT weights, and no sensitivity analysis was carried out to examine the effect of varying charge magnitudes or standoff distances. These factors limit the reliability of the conclusions and emphasize the need for further research involving large-scale testing and parametric studies.

## Conclusion

This study investigated the influence of chemically treated groundnut shell fibers on the mechanical properties and simulated blast performance of high-performance concrete. Results demonstrated that untreated or pretreated fibers generally reduced compressive, split tensile, and flexural strength, while post-treatment with NaOH significantly improved performance at optimized dosages. The most favorable outcomes are quoted as follows.

(i) *Effect of fiber addition without treatment*: Incorporation of untreated or pretreated groundnut shell fibers generally reduced workability, compressive strength, split tensile strength, and flexural strengths at all curing ages.
(ii) *Role of chemical treatment*: Post-treatment with NaOH enhanced the fiber–matrix interaction and improved the mechanical performance of concrete compared to untreated or pretreated fibers.
(iii) *Optimum fiber dosage*: The most favorable mechanical properties were observed at 0.5% post-treated fiber content, which improved compressive, split tensile, and flexural strengths by approximately 10–11% over the control mix at 28 days. Higher fiber contents (≥ 1.0%) led to strength reductions, indicating a clear dosage limit.
(iv) *Blast simulation insights*: Finite element analysis (FEA) of blast loading showed that treated fiber-reinforced concrete panels exhibited better stress distribution and reduced localized deformation compared to conventional concrete. These results are simulation-based and not validated through experimental blast testing.
(v) *Fiber morphology and chemical structure*: FTIR and XRD analyses confirmed that NaOH treatment enhanced the availability of hydroxyl groups and improved crystallinity in the fibers, leading to better bonding potential with the cement matrix.
(vi) *Water absorption and hydration influence*: Post-treated fibers acted as internal curing agents by gradually releasing absorbed water, which helped reduce autogenous shrinkage and improved hydration in high-strength concrete mixes.
(vii) *Effect of excessive fiber content*: At higher dosages (≥ 1.5%), both pretreated and post-treated fibers led to significant reductions in mechanical strength due to higher water absorption and poor dispersion, highlighting the importance of optimized fiber content.

## Limitations of the study

This study has several limitations that should be acknowledged. First, the blast resistance findings are based solely on finite element simulations, as no full-scale experimental blast testing was conducted. Therefore, the predictive results may not fully represent actual structural response under real blast loading. Second, the sample size of laboratory specimens was limited, and no tests were performed on full-scale structural elements, which restricts the generalization of the results. Third, while chemically treated natural fibers improved mechanical performance in the short term, their long-term durability in the alkaline concrete environment, especially under varying temperature and moisture conditions, was not investigated. Finally, the blast simulations considered a limited range of TNT weights without performing a systematic sensitivity analysis, which may overlook variations in response under different charge magnitudes and standoff distances. Both short-term durability tests (e.g., fiber tensile strength after alkaline immersion or curing) and long-term durability studies (e.g., fiber degradation in alkaline matrix, resistance to wet–dry cycles, freeze–thaw, and environmental exposure) are necessary to validate the long-term applicability of groundnut shell fiber reinforced concrete. Future research will therefore focus on systematic durability assessments alongside full-scale blast experiments to ensure practical reliability of this eco-friendly composite. These limitations highlight the need for extended experimental testing, durability assessment, and parametric analysis before field application.

## Implications and future work

- Chemically treated groundnut shell fibers show promise as a sustainable additive to enhance toughness and energy absorption capacity in concrete.
- Further large-scale mechanical tests, durability studies (both long term and short term), and experimental blast trials are required before confirming their suitability for high-impact or blast-resistant structural applications.

## Data Availability

The datasets during and/or analyzed during the current study are available from the corresponding author upon reasonable request.
